# Supplementary material for: Subthalamic nucleus gamma activity increases not only during movement but also during movement inhibition
Source: eLife. 2017 Jul 25;6:e23947. doi: 10.7554/eLife.23947 (PMC5526662; doi:10.7554/eLife.23947)
Supplement: Source code 1. — DOI: http://dx.doi.org/10.7554/eLife.23947.022 [file elife-23947-code1.zip › matlab_code/Read_me.docx]

The main function **run_analyses()** calls all other functions located in **..\code\**. Figures will be saved to **\plots** and .mat-files with the time-frequency decomposition of the data will be stored in **\matfiles**.
The program expects the LFP/EEG data to be located in **..\data\**. The data with the title “**Subthalamic nucleus activity during stopping of rhythmic finger tapping”** can be downloaded from the Oxford Research Archive here:
<https://ora.ox.ac.uk/objects/uuid:54c00c3d-1809-4a52-bba8-b491b6075f35>

You also need to download the fieldtrip toolbox and add the path to your working directory:
<https://github.com/fieldtrip/fieldtrip>
to get access to the following functions:
**ft_preproc_highpassfilter()**, **ft_preproc_lowpassfilter()**

The color schemes used to create the plots have to be downloaded here:
<https://uk.mathworks.com/matlabcentral/fileexchange/34087-cbrewer---colorbrewer-schemes-for-matlab>

The code\ folder also includes the function shadedErrorBar by Rob Campbell:
<http://uk.mathworks.com/matlabcentral/fileexchange/26311-raacampbell-shadederrorbar>

and jbfill by John Bockstege:
<https://uk.mathworks.com/matlabcentral/fileexchange/13188-shade-area-between-two-curves?focused=3777454&tab=function>

**Information to the data:**

The data was collected between 2015 and 2016 at the John Radcliffe Hospital (Oxford) and the National Hospital for Neurology and Neurosurgery (London) in 9 patients with Parkinson's disease who had undergone deep brain stimulation surgery. Patients performed a stopping task while local field potentials from the subthalamic nucleus and scalp EEG channels (C3, C4, Cz, Fz, Pz) were recorded with a TMSi Porti amplifier.

Patients were instructed to interrupt finger tapping, which was paced by a metronome, in response to an auditory stop-signal, which was timed such that successful stopping would occur only in ~50% of all trials. Behavioural data was collected with a goniometer and pressure sensor.

SSMT – stopping condition (main condition: stop signal metronome task)
SSMT.wvData contains the sampleRate (1000, 0.5 Hz high-pass filtered) and recorded waveforms:
'contSTN', 'ipsiSTN', 'contM1', 'ipsiM1' 'Cz', 'Fz', 'Pz',
i.e. contralateral and ipsilateral STN LFP, contralateral and ipsilateral C3/C4 EEG, Cz, Fz, Pz
Movement data was recorded with a goniometer (‘Goni’), pressure sensor (‘Pres’) and EMGs on the FDI muscle (‘FDI’)

SSMT.evt contains behavioural variables that were extracted from the goniometer sensor:

SSMT.evt.movementExtent: Degree of downward movement, i.e. the reverse of stopping success, for each stopping event

SSMT.evt.succes_Stop: Successful stop trials, 0 if the pressure sensor registered a tap, otherwise 1
SSMT.evt.outlier: Behavioural outliers for each stopping event

SSMT.evt.sound_times: Timing of all metronome sounds (in seconds)
SSMT.evt.taps_times: Timing of all taps as measured by the pressure sensor (in seconds)

SSMT.evt.lastRegular_tap_time: Timing of all last regular taps (i.e. the last tap before a stop signal)

SSMT.evt.lastRegular_tap_idx: Array index of all taps (taps_times) that were last regular taps

SSMT.evt.stopSig_times: Timing of all stop signals

SSMT.evt.RT: Time difference between taps and the corresponding closest metronome sound, during sensorimotor synchronization this becomes negative as the tap slightly precedes the sound (see literature on tapping and “negative mean asynchrony”)
SSMT.evt.taps_nr: Number of tap within each trial (1-9)

SSMT.evt also contains fields that store trials that were classified as outliers in the waveform data, e.g. EEG or LFP artefacts:
SSMT.evt.lastTap_EEG_outl: Outliers removed regarding specifically the end of each trial at the time of the last tap and stop signal

SSMT.evt.allTapss_EEG_outl: Outliers removed regarding all regular taps

CTMT – Control condition (continue tapping metronome task), here participants did not attempt to stop but continued after the stop signal with two more taps, structured in the same way as the SSMT structure.
